# Pharmacodynamics of Antimicrobials against *Mycoplasma mycoides mycoides* Small Colony, the Causative Agent of Contagious Bovine Pleuropneumonia

**DOI:** 10.1371/journal.pone.0044158

**Published:** 2012-08-27

**Authors:** John D. Mitchell, Quintin A. McKellar, Declan J. McKeever

**Affiliations:** 1 Royal Veterinary College, Hatfield, Hertfordshire, United Kingdom; 2 University of Hertfordshire, Hatfield, Hertfordshire, United Kingdom; Auburn University, United States of America

## Abstract

**Background:**

*Mycoplasma mycoides* subspecies *mycoides* Small Colony (*Mmm*SC) is the causative agent of Contagious Bovine Pleuropneumonia (CBPP), a disease of substantial economic importance in sub-Saharan Africa. Failure of vaccination to curtail spread of this disease has led to calls for evaluation of the role of antimicrobials in CBPP control. Three major classes of antimicrobial are effective against mycoplasmas, namely tetracyclines, fluoroquinolones and macrolides. Therefore, the objectives of this study were to determine the effector kinetics of oxytetracycline, danofloxacin and tulathromycin against two *Mmm*SC field strains in artificial medium and adult bovine serum.

**Methods:**

Minimum inhibitory concentrations (MIC) were determined for oxytetracycline, danofloxacin and tulathromycin against *Mmm*SC strains B237 and Tan8 using a macrodilution technique, and time-kill curves were constructed for various multiples of the MIC over a 24 hour period in artificial medium and serum. Data were fitted to sigmoid E_max_ models to obtain 24 hour-area under curve/MIC ratios for mycoplasmastasis and, where appropriate, for mycoplasmacidal activity and virtual mycoplasmal elimination.

**Results:**

Minimum inhibitory concentrations against B237 were 20-fold higher, 2-fold higher and approximately 330-fold lower in serum than in artificial medium for oxytetracycline, danofloxacin and tulathromycin, respectively. Such differences were mirrored in experiments using Tan8. Oxytetracycline was mycoplasmastatic against both strains in both matrices. Danofloxacin elicited mycoplasmacidal activity against B237 and virtual elimination of Tan8; similar maximum antimycoplasmal effects were observed in artificial medium and serum. Tulathromycin effected virtual elimination of B237 but was mycoplasmastatic against Tan8 in artificial medium. However, this drug was mycoplasmastatic against both strains in the more physiologically relevant matrix of serum.

**Conclusions:**

Oxytetracycline, danofloxacin and tulathromycin are all suitable candidates for further investigation as potential treatments for CBPP. This study also highlights the importance of testing drug activity in biological matrices as well as artificial media.

## Introduction

*Mycoplasma mycoides* subspecies *mycoides* Small Colony (*Mmm*SC) is the causative agent of Contagious Bovine Pleuropneumonia (CBPP), a major trans-boundary disease of livestock in sub-Saharan Africa [1] and an enduring threat to cattle in Europe. Indeed, CBPP is listed by the World Organisation for Animal Health as one of the diseases that it monitors on a global scale. The disease impacts farmers directly through mortality and poor productivity of their animals, and indirectly through missed opportunities for trade due to import bans and quarantine [1]. Contagious bovine pleuropneumonia manifests as a range of syndromes, from acute pneumonia to a protracted chronic phase, during which apparently recovered animals can harbour and shed the pathogen [2]. *Mmm*SC is spread by aerosol, primarily over only short distances [3], and therefore movement of cattle plays a key role in dissemination of the disease. Since livestock movement patterns in Africa are complex and difficult to control [4], state veterinary services have largely relied upon the use of live attenuated vaccines, in particular the T_1_/44 strain, to control the disease. However, these are constrained by cold chain dependence, short-lived immunity and adverse events, including inoculation site reactions and occurrence of mild forms of the disease itself [5].

Several reports have suggested that antimicrobial therapy can improve the outcome of infection [6], [7], [8], [9] and recent modelling studies suggest that co-deployment of antimicrobials would substantially enhance the impact of vaccination campaigns [4]. However, the use of antimicrobials in CBPP control has been discouraged largely because of the view that it favours the creation of chronic carriers [2]. Although there is no hard evidence for this, the use of antimicrobials to treat CBPP is not permitted in several countries where the disease is prevalent. Nevertheless, three major classes of antimicrobial are effective against mycoplasmas, namely tetracyclines, fluoroquinolones and macrolides [10]. Indeed, several antimicrobials have emerged on to the market since 1987, some of which show mycoplasmacidal activity *in vitro* [11], [12]. The *in vivo* efficacy of such antimicrobials against *Mmm*SC remains to be fully determined, as studies have been few. In naturally affected cattle, treatment with danofloxacin, a fluoroquinolone, significantly reduced transmission of *Mmm*SC to healthy in-contact cattle but failed to improve the clinical outcome [9]. In contrast, long-acting oxytetracycline (OTC) improved clinical condition and, like danofloxacin, also prevented disease transmission to in-contact cattle; however, a bacteriological cure was not achieved in all treated animals [6].

Such partial efficacy may arise from sub-optimal dosage regimens. Increasingly, pharmacokinetic-pharmacodynamic (PK-PD) modelling is employed in veterinary medicine to determine antimicrobial dosages that result in bacteriological eradication, thereby reducing the risk of persistent carrier infections and development of resistance [13]. Indeed, such an approach led to a revision by the manufacturer of the dosage recommended for danofloxacin in the treatment of calf pneumonia [14]. As the first step towards defining dosage strategies for the treatment of CBPP, we investigated the *in vitro* killing profiles of OTC, danofloxacin and tulathromycin (tetracycline, fluoroquinolone and triamilide macrolide, respectively) against the virulent Kenyan *Mmm*SC strain B237 in artificial medium to assess antimicrobial efficacy. Because the composition of artificial medium differs from that of biological matrices, so that minimum inhibitory concentration (MIC) may differ between the two, we extended these studies to adult bovine serum, as a proxy for the ‘shallow biophase,’ namely plasma, interstitial fluid and well perfused tissues [15], which are the sites of most bacterial infections [16]. To determine whether findings were replicable in another *Mmm*SC strain, antimicrobial killing profiles in artificial medium and adult bovine serum were established for the Tanzanian field strain Tan8 [17]. We present preliminary pharmacodynamic modelling analysis of the data arising from these studies. Our observations are consistent with variation in efficacy between the drugs and illustrate the importance of evaluating drug function in biological matrices as well as artificial medium.

## Materials and Methods

### Materials

*Mmm*SC strains B237, originally isolated in Kenya [18], and Tan8 were provided by Joachim Frey, University of Bern, Switzerland, and the Animal Health and Veterinary Laboratories Agency, Weybridge, UK, respectively. Oxytetracycline hydrochloride and danofloxacin were obtained from Sigma-Aldrich (VETRANAL^TM^, Poole, UK), and tulathromycin was supplied by Pfizer Ltd. (Kalamazoo, Michigan, US). Oxytetracycline hydrochloride was dissolved in double distilled water, danofloxacin in 0.01 M sodium hydroxide and tulathromycin in 0.0015 Mcitric acid. Artificial liquid and solid media were obtained from Mycoplasma Experience (Reigate, UK) and adult bovine serum from Sigma-Aldrich (Poole, UK). Liquid medium contained the pH indicator phenol red and was adjusted to pH 7.6 such that the indicator was red in colour.

### Determination of MIC

Initially, MIC was determined using a microdilution technique, as described by Hannan [19]. To prepare the inoculum, mycoplasma were subcultured in liquid medium and diluted while in exponential phase to give the desired inoculum size. Briefly, series of doubling dilutions were prepared for each antimicrobial (final concentration ranges of 2×10^−3^ –8 mg/L for OTC and tulathromycin, and 0.03–8 mg/Lfor danofloxacin) in liquid medium. Triplicate 0.1 mL aliquots of each antimicrobial concentration were transferred to wells of a 96-well plate and supplemented with 0.1 mL of *Mmm*SC strain B237 diluted in liquid medium to give a final titre of 10^7^ cfu/mL, the intended initial titre for subsequent time-kill studies. Growth controls (*Mmm*SC in absence of antimicrobials), end-point controls (blank medium set at pH 6.8), solvent controls (*Mmm*SC in presence of solvent at the concentration used to dissolve antimicrobials) and sterility controls (blank medium) were also included. After sealing with gas permeable film, plates were incubated at 37°C and assessed visually for any change in indicator colour at least three times daily until that of the growth control matched that of the end-point control. Minimum inhibitory concentration was defined as the lowest concentration of antimicrobial to show no change in the colour of the indicator. Since the error associated with establishing an MIC on doubling dilutions can be up to virtually 100% [20], five sets of overlapping doubling dilutions were used to define the MIC with greater accuracy.

For comparison, MICs were also determined by a macrodilution technique using volumes of 4 mL across the same range of antimicrobial concentrations and with an initial mycoplasmal titre of 10^7^ cfu/mL. Cultures were incubated for 24 hours at 37°C and, at 0 and 24 hours, samples were removed and serially diluted ten-fold down to 10^−5^. Aliquots of each dilution were transferred to solid medium and incubated at 37°C in a humidified atmosphere of 5% carbon dioxide in air for at least 4 days. Minimum inhibitory concentration was defined as the lowest concentration of antimicrobial that prevented any increase in mycoplasma cfu/mL over the incubation period. Minimum inhibitory concentrations were also determined at initial inoculum sizes of 10^4^, 10^5^ and 10^6^ cfu/mL to ascertain whether there was any inoculum effect (IE). For MIC determination in serum, five overlapping sets of doubling dilutions of antimicrobial were prepared and inoculated with exponential phase culture, such that the mycoplasmal titre was 10^6^ cfu/mL in 2 mL volumes of 99∶1 (v/v) serum:artificial medium.

### Time-kill studies

Inocula for time-kill studies were prepared as described above. Liquid medium containing various concentrations of antimicrobial, which corresponded to multiples of the MICs given by the macrodilution technique, was inoculated with *Mmm*SC strain B237 to achieve an initial titre of 10^7^ cfu/mL in a volume of 4 mL. Cultures were incubated at 37°C for 24 hours and, at 0, 2, 4, 8 and 24 hours, samples were removed and serially diluted down to 10^−5^. Aliquots (10 µL) of each dilution were transferred to solid medium and incubated at 37°C in a humidified atmosphere of 5% carbon dioxide in air. Colonies were generally counted using the dilution which gave between 30 and 300 colonies per plate and values were converted to cfu/mL. Antimycoplasmal effect, defined as the change in log_10_ (cfu/mL) over a 24 hour time period, was determined for each concentration of antimicrobial in both matrices on at least two occasions. This was done to ensure reproducibility and data were averaged for subsequent pharmacodynamic analysis. The limit of detection was 2 log_10_ (cfu/mL) units. Parallel studies were conducted in the absence of antimicrobials and in the presence of solvents at concentrations used to dissolve antimicrobials for control purposes. Time-kill studies in adult bovine serum were performed as for those in artificial medium, except that initial titres of 10^6^ cfu/mL in 2 mL volumes of serum were used. Initial inoculum size was smaller than in studies using artificial medium as *Mmm*SC attained a maximum titre of only 10^7^ cfu/mL in serum. For comparison, studies to determine MIC (macrodilution technique with a single set of doubling dilutions) and time-kill assays were repeated using *Mmm*SC strain Tan8 in artificial medium and adult bovine serum.

### Pharmacodynamic analysis

Twenty-four hour-area under curve (AUC):MIC ratios were calculated for each antimicrobial concentration. Using Phoenix WinNonlin 6.2 professional software (Pharsight Corporation, Mountain View, CA, USA), data were subsequently fitted to a sigmoid E_max_ model given by the equation,
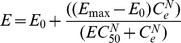
where E is the antimycoplasmal effect, E_0_ is the difference in log_10_ (cfu/mL) after 24 hours compared to the initial titre when no antimicrobial is present, E_max_ is the maximum antimycoplasmal effect, EC_50_ is the AUC:MIC ratio for an antimicrobial that gives rise to 50% of the maximal response, C_e_ is the AUC:MIC ratio of the antimicrobial in the effect compartment (i.e. artificial medium or serum) and N is the Hill coefficient, which reflects the slope of the relationship between antimycoplasmal effect and AUC:MIC. From the resulting graph, AUC:MIC ratios were obtained for mycoplasmastatic (E = 0, no change in mycoplasmal count after 24 hours) and mycoplasmacidal (E = −3, 99.9% reduction of original inoculum count after 24 hours) activity of antimicrobials, and for virtual mycoplasmal elimination (E = −4, 99.99% reduction of original inoculum count after 24 hours).

## Results

### Determination of MIC for antimicrobials against MmmSC strain B237

**Figure 1:**
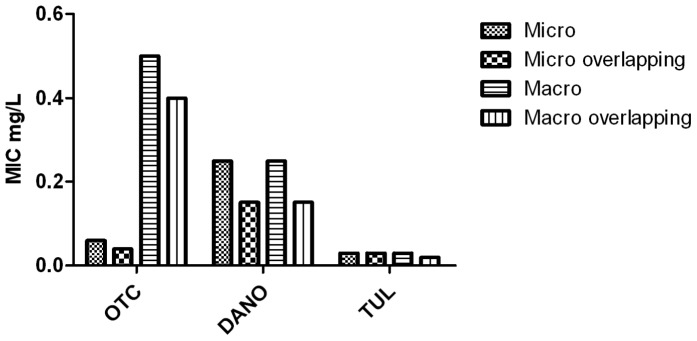
Minimum inhibitory concentrations. Minimum inhibitory concentrations for oxytetracycline (OTC), danofloxacin (DANO) and tulathromycin (TUL) against *Mmm*SC strain B237 in artificial medium using microdilution and macrodilution techniques (inoculum size 10^7^ cfu/mL). Values were based on either just one set of doubling dilutions (micro, macro) or five overlapping sets of doubling dilutions (micro overlapping, macro overlapping).

Minimum inhibitory concentration values obtained for danofloxacin and tulathromycin in artificial medium were comparable using microdilution and macrodilution techniques (figure 1). However, the MIC value obtained for OTC by microdilution was approximately ten-fold lower than that obtained by macrodilution. This suggested that visual inspection for a change in indicator colour was not sufficiently sensitive to detect small increases in mycoplasmal growth. Minimum inhibitory concentrations obtained using the macrodilution technique were therefore used for subsequent studies. These were 0.40, 0.15 and 0.02 mg/L for OTC, danofloxacin and tulathromycin, respectively. Errors arising from the use of a single set of doubling dilutions for establishing MIC values were 25%, 67% and 50% for OTC, danofloxacin and tulathromycin, respectively, highlighting the importance of using overlapping dilution series.

**Figure 2:**
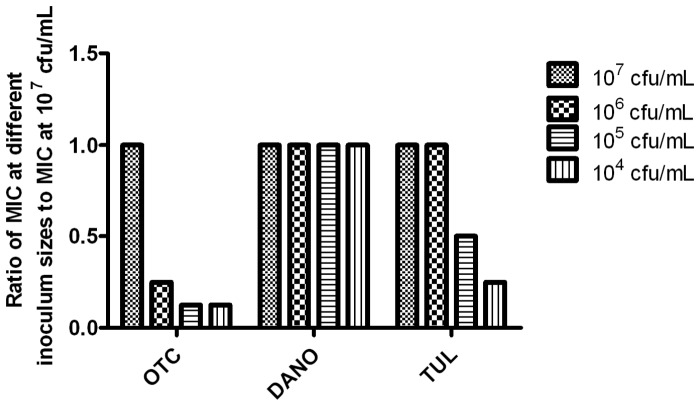
Effect of inoculum size on MIC. Ratio of MIC at different inoculum sizes to MIC at 10^7^ cfu/mL for oxytetracycline (OTC), danofloxacin (DANO) and tulathromycin (TUL) against *Mmm*SC strain B237 in artificial medium.

Minimum inhibitory concentrations were also determined at different inoculum sizes to evaluate the effect of this parameter on the MIC value obtained (figure 2). Whereas IEs were observed for tulathromycin and OTC, the largest of which was an 8-fold increase in MIC for OTC when inoculum size increased from 10^5^ to 10^7^ cfu/mL, no IE was evident for danofloxacin.

Minimum inhibitory concentrations determined in adult bovine serum were 2.0 mg/L, 0.3 mg/L and 0.06 µg/L for OTC, danofloxacin and tulathromycin, respectively. These values were 20-fold higher (OTC), 2-fold higher (danofloxacin) and approximately 330-fold lower (tulathromycin) than those determined in artificial medium at the equivalent inoculum size of 10^6^ cfu/mL.

### Time-kill curves and pharmacodynamic analysis

**Figure 3:**
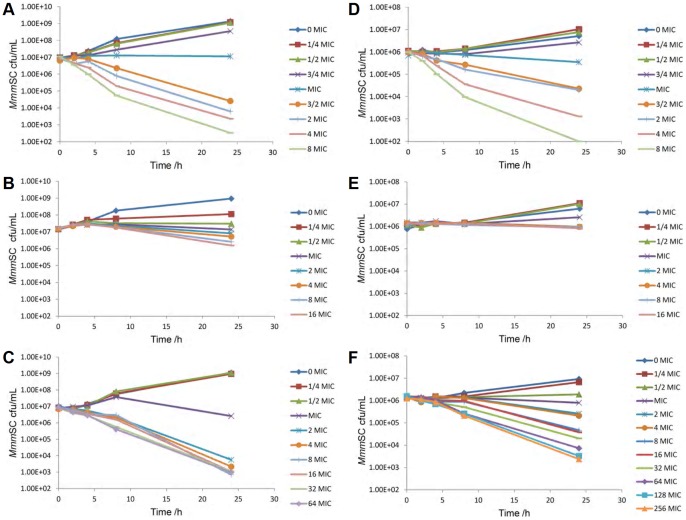
Time-kill curves. Representative time-kill curves for (A) danofloxacin, (B) oxytetracycline and (C) tulathromycin in artificial medium, and (D) danofloxacin, (E) oxytetracycline and (F) tulathromycin in adult bovine serum against *Mmm*SC strain B237.

**Figure 4:**
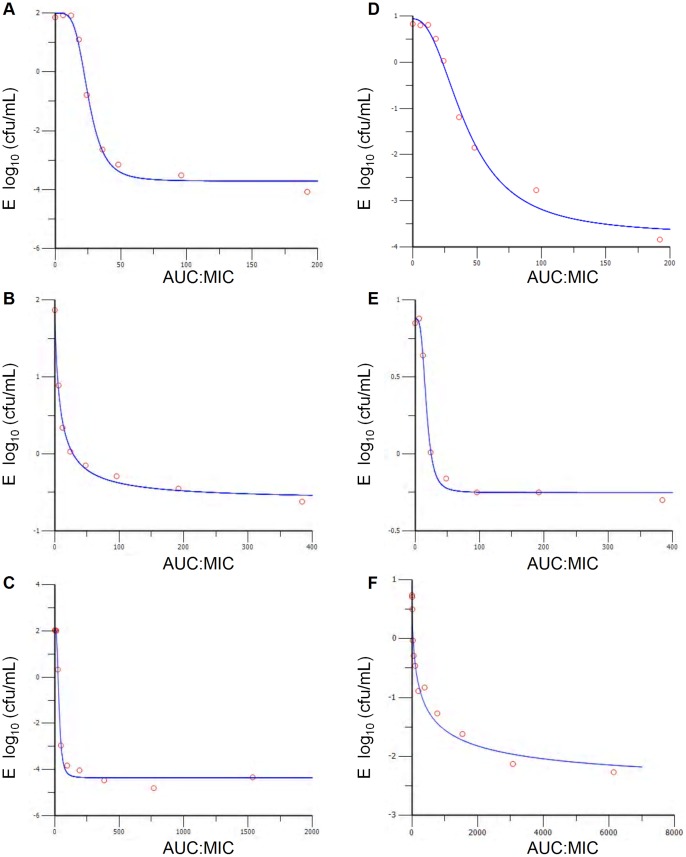
Sigmoid E_max_ models. Sigmoid E_max_ relationships for antimycoplasmal effect (E, log_10_ (cfu/mL)) versus *in vitro* AUC:MIC ratio, derived from data obtained from time-kill curves for (A) danofloxacin (B) oxytetracycline and (C) tulathromycin in artificial medium, and (D) danofloxacin, (E) oxytetracycline and (F) tulathromycin in adult bovine serum against *Mmm*SC strain B237.

Representative graphs of the *in vitro* killing profiles of OTC, danofloxacin and tulathromycin against *Mmm*SC strain B237 in artificial medium and adult bovine serum are presented in figure 3, with sigmoid E_max_ models shown in figure 4. Twenty-four hour-AUC:MIC ratios for ycoplasmastasis and, where appropriate, mycoplasmacidal activity and virtual mycoplasmal elimination are provided in table 1. Oxytetracycline had a mycoplasmastatic action in artificial medium, eliciting a maximum antimycoplasmal effect of only −0.61 log_10_ (cfu/mL) units (table 1). Danofloxacin was mycoplasmacidal with a maximum antimycoplasmal effect of −3.71 log_10_ (cfu/mL) units and tulathromycin elicited virtual mycoplasmal elimination with a maximum antimycoplasmal effect of −4.36 of log_10_ (cfu/mL) units. The presence of solvents at concentrations used to dissolve antimicrobials had no effect on mycoplasmal growth.

**Table 1:** Pharmacodynamic analysis of data obtained from *in vitro* time-kill studies in artificial medium and adult bovine serum for oxytetracycline (OTC), danofloxacin (DANO) and tulathromycin (TUL) against *Mmm*SC strain B237.

	Artificial Medium			Adult Bovine Serum		
Variable	DANO	OTC	TUL	DANO	OTC	TUL
E_0_	2.00	1.88	2.12	0.94	0.88	0.88
E_max_	−3.71	−0.61	−4.36	−3.74	−0.25	−2.63
MS	21.80	28.36	26.00	23.12	24.80	35.00
MC	39.78	-	50.01	86.63	-	-
VME	-	-	81.01	-	-	-
EC_50_	25.23	8.29	32.69	42.04	17.41	239.99
N	4.27	0.92	3.11	2.31	3.53	0.57

E_0_ is the difference in log_10_ (cfu/mL) units after 24 hours compared to the initial titre when no antimicrobial is present, E_max_ is the maximum antimycoplasmal effect in log_10_ (cfu/mL) units, EC_50_ is the twenty-four hour-area under curve:minimum inhibitory concentration ratio (AUC:MIC) of antimicrobial that gives rise to 50% of the maximum response and N is the Hill coefficient. AUC:MIC ratios are provided for mycoplasmastatic activity (MS) and, where appropriate, mycoplasmacidal activity (MC) and virtual mycoplasmal elimination (VME).

As in artificial medium, OTC was mycoplasmastatic and danofloxacin was mycoplasmacidal in adult bovine serum, producing maximum antimycoplasmal effects of −0.25 and −3.74 log_10_ (cfu/mL) units, respectively. However, in marked contrast to artificial medium, tulathromycin was mycoplasmastatic in serum with a maximum antimycoplasmal effect of only −2.63 log_10_ (cfu/mL) units.

### Killing profiles of antimicrobials against MmmSC strain Tan8

Using just one set of doubling dilutions to define MIC, values for danofloxacin, OTC and tulathromycin against *Mmm*SC strain Tan8 were 0.125, 0.25 and 0.016 mg/L in artificial medium, and 0.5, 1 and 3×10^−5^ mg/L in adult bovine serum. Danofloxacin elicited virtual mycoplasmal elimination in both matrices (E_max_, log_10_ (cfu/mL) units: −4.08, artificial medium; −4.19, serum), while OTC (E_max_, log_10_ (cfu/mL) units: −1.22, artificial medium; −1.09, serum) and tulathromycin (E_max_, log_10_ (cfu/mL) units: −2.80, artificial medium; −0.72, serum) were mycoplasmastatic in both.

## Discussion

A large swathe of antimicrobials can be ruled out for the treatment of diseases caused by mycoplasmas because they lack a cell wall. Nevertheless, the fluoroquinolones, tetracyclines and macrolides have shown efficacy against these organisms [10], although few studies have specifically addressed the potential of these drugs for the treatment of CBPP. The aim of the present study was to determine the killing kinetics of three antimicrobial agents against the virulent Kenyan B237 strain of *Mmm*SC in both artificial medium and adult bovine serum. This forms part of a longer term goal to use the PK-PD modelling approach outlined by Lees *et al*. [21] to determine optimised dosage strategies for the treatment of this disease. The approach has a major advantage over classical dose titration studies; whereas dose titration is generally focused on clinical outcome and is not informative of bacteriological cure, PK-PD modelling allows optimisation of antimicrobial dose towards a desired bacteriological outcome, reducing the risks of persistent carrier infections and development of resistance. Indeed, dose titration studies are confounded by the ‘Pollyanna effect,’ whereby the efficacy of a drug giving a good bacteriological response is often under-estimated while that of a drug with a poor bacteriological response is over-estimated [15], [22].

Danofloxacin, OTC and tulathromycin were selected as representatives of the three classes of antimicrobial known to have efficacy against mycoplasmas. Minimum inhibitory concentrations were initially determined for each drug against *Mmm*SC strain B237 to define a concentration range over which time-kill assays should be performed. To date, MICs have been published for only OTC and danofloxacin against a number of *Mmm*SC isolates, with MIC ranges of 0.125–4 mg/L and <0.06–0.5 mg/L for OTC and 0.125–1 mg/L and 0.12–0.5 mg/L for danofloxacin in artificial medium [11], [12]. Minimum inhibitory concentrations against *Mmm*SC have not been reported for tulathromycin, although Godinho *et al*. [23] reported MICs of <0.004–0.125 mg/L against *Mycoplasma hyopneumoniae* isolates. However, because standardised conditions are lacking for *in vitro* susceptibility testing of veterinary mycoplasmas, the MIC is potentially a crude measure of antimicrobial activity. Indeed, MIC can vary widely between the strains within a species and methodological factors, such as medium composition, inoculum size and reading of the test, can influence the value obtained [24]. Minimum inhibitory concentrations obtained from the present study are therefore not directly comparable with those from previous studies.

Minimum inhibitory concentrations were determined using both microdilution and macrodilution techniques. Whereas the latter is based on post-incubation changes in colony counts relative to the original inoculum, the former relies upon visual assessment of pH indicator colour change to represent mycoplasmal growth. Although these two methods produced comparable MIC values for danofloxacin and tulathromycin, the MIC for OTC was ten-fold lower when the microdilution technique was followed, suggesting that visual observation of a colour change was not sufficiently sensitive to detect small increases in mycoplasmal growth. It is likely that this issue was only encountered for OTC because both danofloxacin and tulathromycin had steeper relationships between AUC:MIC and antimycoplasmal effect, with Hill coefficients of 4.27 and 3.11 for danofloxacin and tulathromycin, respectively, versus 0.92 for OTC.

Inoculum size can also have considerable impact on MIC and the IE, defined as a significant increase in the MIC of an antimicrobial when the number of organisms inoculated is increased [25], has been well documented for some combinations of antimicrobial and mycoplasma species [19]. Although recommendations state that MIC should be determined at a mycoplasma inoculum of 10^3^–10^5^ cfu/mL [19], MICs were initially established at 10^7^ cfu/mL in artificial medium; this was the intended initial titre for time-kill assays, which needed to be sufficiently high to demonstrate maximal antimycoplasmal effects of drugs. In fact, it can be speculated that a larger inoculum size more closely resembles the bacterial density at the site of infection [26]. However, when MICs were determined at smaller inoculum sizes, a significant IE (i.e. ≥8-fold rise in MIC at a higher inoculum compared to a lower inoculum) was observed for OTC, although no effect was evident for danofloxacin. This is consistent with previous observations that inoculum size has a less dramatic effect on MIC for fluoroquinolones than for than for other antimicrobial classes [27], [28]. Nonetheless, a large body of evidence suggests that the occurrence and magnitude of an IE depend on the combination of pathogen and antimicrobial under scrutiny [25]. Inoculum size may also influence bactericidal activity. For example, Morrissey and George [29] showed that as inoculum size of *Streptococcus pneumoniae* was increased, bactericidal activity of fluoroquinolones declined to the extent that they were only bacteriostatic at 10^13^ cfu/L. The clinical relevance of IE is controversial. While Craig *et al*. [30] refute that *in vitro* inoculum effects have a bearing on *in vivo* antimicrobial efficacy, at least for β-lactams, Soriano *et al*. [31] found that higher serum concentrations relative to the MIC were required to reduce mortality of rats infected with *Escherichia coli* for drugs with a pronounced IE when compared to those with little or no IE. Furthermore, higher effective doses of fluoroquinolones and, to a greater extent, carbapenems were observed in mice infected with large inocula of either *Staphylococcus aureus* or *Pseudomonas aeruginosa* compared to those infected with smaller inocula [32]. Finally, an IE may result in over-estimation of the dosage by PK-PD modelling approaches, particularly if the *in vivo* inoculum size is smaller than that used in *in vitro* studies [33].

Minimum inhibitory concentrations were also affected by matrix composition, as highlighted by the differences observed in artificial medium and bovine serum. Minimum inhibitory concentrations for danofloxacin and OTC were respectively two-fold and 20-fold greater in serum than artificial medium at an inoculum size of 10^6^ cfu/mL. Since only the free fraction of drugs is active in plasma and total protein concentration in serum was higher than in artificial medium (data not shown), the higher MICs in serum may be explained, at least in part, by protein binding. In this regard, danofloxacin and OTC exhibit plasma protein binding of 49% [34] and 71.7% [35], respectively. In addition, the calcium ion concentration was higher in serum than in artificial medium and it can be speculated that this resulted in increased chelation of OTC in serum [36]. In marked contrast to danofloxacin and OTC, the MIC of tulathromycin was approximately 330-fold lower in serum. Such a low MIC (0.06 µg/L) is not unprecedented; Devine and Hagerman [37] reported an MIC of 0.1 µg/L for coumermycin A_1_ against *Neisseria meningitidis*. The potency of the weak base tulathromycin in acidic conditions is expected to be lower due to ionisation and the consequent inability to penetrate cell membranes. It has been shown previously that acidification of culture medium with carbon dioxide resulted in higher MICs for tulathromycin against *Actinobacillus pleuropneumoniae* and *Haemophilus somnus*, while the presence of serum reduced the MICs [38]. It was noted that the pH of adult bovine serum and also fresh sterile-filtered calf serum was higher than that of artificial medium (approximately pH 8 versus pH 7.6). The MIC of tulathromycin against *Mmm*SC in artificial medium set at pH 8 was 0.008 mg/L at a 10^6^ cfu/mL inoculum size, i.e. 2.5-fold lower than at pH 7.6 (data not shown). Such a pH difference may therefore contribute, at least in part, to the MIC difference observed between artificial medium and adult bovine serum. In addition, it is plausible that acidification arising from the release of carbon dioxide from *Mmm*SC resulted in reduced drug potency in artificial medium. Under these circumstances, lower MIC values observed in serum would arise from its natural buffering capacity. However, despite this apparent enhanced potency and the smaller inoculum size used for time-kill assays, tulathromycin was only mycoplasmastatic in this matrix, whereas it elicited virtual mycoplasmal eradication in artificial medium. This conflicts with previous reports of serum enhancement of the antibacterial effect of tulathromycin against *H. somnus* and a shortening of the time to exert bactericidal effects against *A. pleuropneumoniae* [38]. However, we have observed that tulathromycin elicits only mycoplasmastatic effects against lag phase *Mmm*SC in artificial medium (data not shown). The observed discrepancy between serum and artificial medium may therefore relate to the longer lag phase that occurs in serum after mycoplasma culture is diluted to obtain the desired inoculum size for time-kill assays.

Although the aim of the current study was to provide antimicrobial dosage protocols specifically for cattle infected with *Mmm*SC strain B237, MIC and time-kill assays were repeated using the Tanzanian field strain Tan8 to determine whether the effects observed were replicable in a different strain. The MIC values (based on one set of doubling dilutions) against B237 and Tan8 were comparable, being at most only two-fold lower against Tan8. In addition, the differences between MIC values obtained in artificial medium and serum against Tan8 mirrored those observed against B237. Regarding antimicrobial activity, OTC behaved similarly against B237 and Tan8, as did danofloxacin, with differences of less than 1 log_10_ (cfu/mL) unit between E_max_ values against each strain. However, whereas tulathromycin effected virtual elimination of B237 in artificial medium, it was only mycoplasmastatic against Tan8. Despite this, in the physiologically more relevant matrix of serum, this drug was mycoplasmastatic against both strains.

Pharmacodynamic analysis allows estimations of dosages that would be required *in vivo* for either mycoplasmastatic or mycoplasmacidal activity using the formula,
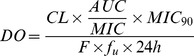
where DO is the optimal dose (mg/kg/day), CL is the body clearance (L/kg/day), AUC/MIC is the breakpoint surrogate marker for the required effect (h), MIC_90_ is the MIC for 90% of strains of a species (mg/L), F is the bioavailability (from 0 to 1) and f_u_ is the free drug fraction (from 0 to 1). This formula can be simplified if time-kill assays are performed in serum as no correction is necessary for protein binding and, in the case of the present study, since we are aiming to provide dosage regimens for cattle infected with the virulent strain B237, MIC_90_ can be replaced by the strain-specific MIC. If the drug can be administered by intravenous (i.v.) route, F can also be excluded [20]. This gives



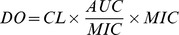
where clearance is expressed as L/kg/h.

Using the AUC:MIC ratios generated in serum and previously published data for body clearance and bioavailability, dosages for mycoplasmastasis can be calculated for OTC and tulathromycin, and a mycoplasmacidal dosage can be estimated for danofloxacin. A meta-analysis of PK data for long-acting OTC gave a clearance of 0.115 L/kg/h [39] and bioavailability has been recorded at 78.5% via the intramuscular route in male cattle [40]. Together with an AUC:MIC ratio of 24.80 and MIC of 2 mg/L in serum, a predicted dosage of 7.3 mg/kg/day is obtained for mycoplasmastasis of strain B237. Based on an AUC:MIC ratio of 26.40 and MIC of 1 mg/L in serum, a lower dosage of 3.9 mg/kg/day is obtained for mycoplasmastasis of strain Tan8. Both are below the licensed dose of 20 mg/kg. It is believed that OTC is one of the most widely used drugs to treat CBPP in Africa [12]. However, information regarding current dosing practice is not available.

The clearance of tulathromycin in beef calves was 0.181 L/kg/h and the bioavailability was 91.3% by the subcutaneous (s.c.) route [41]. Given an AUC:MIC ratio of 35.00 and an MIC of 0.06 µg/L, a dosage of at least 0.4 µg/kg/day is estimated to achieve mycoplasmastasis of strain B237. To obtain the same effect against Tan8, a dosage of 0.15 µg/kg/day is predicted to be required (AUC:MIC ratio, 24.88; MIC, 3×10^−5^ mg/L). Again both are far below the licensed dosage of 2.5 mg/kg. Furthermore, the *in vivo* distribution of this drug is particularly advantageous; lung concentrations have been measured at 11–325 times higher than those concurrently in serum and the half-time for elimination from lung was 184 hours, giving rise to a potentially prolonged exposure of respiratory pathogens to tulathromycin [41]. However, a disadvantage of using this drug to treat CBPP at its current licensed dosage is the contra-indication of treating cattle whose milk is intended for human consumption. However, it may be possible to revise the datasheet for this drug if the lower dosage suggested by the current study can be adopted.

For danofloxacin, bioavailability was 94% if administered via the s.c. route and plasma clearance was 0.468 L/kg/h in cross-bred calves [42]. Given an AUC:MIC ratio of 86.63 for mycoplasmacidal activity and an MIC of 0.30 mg/L, the optimal dosage would be 12.9 mg/kg/day to treat cattle infected with strain B237. A similar dosage of 14.7 mg/kg/day is predicted to elicit mycoplasmacidal activity against strain Tan8 (AUC:MIC ratio, 59.19; MIC, 0.5 mg/L). Furthermore, a dosage of 44.4 mg/kg/day is estimated for virtual elimination of strain Tan8 (AUC:MIC ratio, 178.34; MIC, 0.5 mg/L) but this is over seven-fold greater than the licensed dosage (6 mg/kg, single injection, s.c. or i.v.) and side-effects may be encountered. At the licensed dosage, only mycoplasmastasis would occur. This may explain the lack of effect of 2.5 mg/kgdanofloxacin on the clinical score of CBPP-affected cattle reported by Huebschle *et al* [9]. However, it is important to remember that the PK-PD modelling approach does not take into account the immune response of the animal. This may act additively or synergistically with the antimicrobial agent, and the predicted dosage may therefore be an over-estimate. In addition, a limitation of the dosage prediction stems from the fact that previously published PK data are derived from studies on calves, albeit ruminating in the majority of cases. Although most changes in PK seem to occur between newborn pre-ruminant and ruminating stages [43], [44], it is possible that age-related PK changes exist between calves and adult cattle, as observed in the human population [45]. Furthermore, PK parameters may be influenced by gender [46], physiological status [47], breed of cattle [48], or indeed the species (i.e. *Bos taurus* versus *Bos indicus*).

The results of this study show efficacy for danofloxacin, OTC and tulathromycin against virulent *Mmm*SC strain B237 *in vitro*, providing evidence that all three may be suitable candidates for the treatment of CBPP caused by this strain. Similar observations were made for strain Tan8, suggesting the findings of this study may apply across the species. Although dosages can be estimated from fixed concentration pharmacodynamic models, these do not account for the decline in concentration as drug is cleared from the body. The next stage will be to develop *in vitro* dynamic concentration models [49] to simulate *in vivo* antimicrobial PK, enabling not only determination of post-antibiotic-sub-MIC inhibitory effects of antimicrobials but also assessment of concentration and/or time dependence of antimicrobial activity through administration of drug by bolus and infusion. Finally, this study highlights the importance of susceptibility testing in biological fluids in addition to artificial media, as demonstrated by marked differences in MICs between the two matrices.
